# Polyethylene Oxide (PEO) Provides Bridges to Silica Nanoparticles to Form a Shear Thickening Electrolyte for High Performance Impact Resistant Lithium‐ion Batteries

**DOI:** 10.1002/advs.202302844

**Published:** 2023-08-06

**Authors:** Zhiqi Chen, Yunfeng Chao, Sepidar Sayyar, Tongfei Tian, Kezhong Wang, Yeqing Xu, Gordon Wallace, Jie Ding, Caiyun Wang

**Affiliations:** ^1^ ARC Centre of Excellence for Electromaterials Science Intelligent Polymer Research Institute AIIM Facility Innovation Campus University of Wollongong Wollongong NSW 2500 Australia; ^2^ Australian National Fabrication Facility – Materials Node Innovation Campus University of Wollongong Wollongong NSW 2500 Australia; ^3^ School of Science Technology and Engineering University of the Sunshine Coast Sippy Downs QLD 4556 Australia; ^4^ Platforms Division Defence Science & Technology Group 506 Lorimer Street Fishermans Bend VIC 3207 Australia

**Keywords:** impact tolerance, lithium‐ion batteries, polymer bridging, safety, shear thickening electrolytes

## Abstract

The development of shear thickening electrolytes is proving to be pivotal in the quest for impact resistant lithium‐ion batteries (LIBs). However, the high viscosity and poor stability associated with the need for high filler content has to date impeded progress. Here, this work reports a new type of polymer‐bridged shear thickening electrolyte that overcomes these shortcomings, by utilizing the interaction between polymer chains and silica nanoparticles. The incorporation of polyethylene oxide (PEO) facilitates hydrocluster formation providing impact resistance with a filler content as low as 2.2 wt%. This low viscosity electrolyte has a high ionic conductivity of ≈5.1 mS cm^−1^ with excellent long‐term stability, over 30 days. The effectiveness of this electrolyte in LIBs is demonstrated by excellent electrochemical performance and high impact resistance.

## Graphical abstract

An efficient polymer‐bridged shear thickening electrolyte with low viscosity, high ionic conductivity, and long‐term stability is realized by utilizing the interaction between silica particles and polymer chains. The assembled batteries demonstrate excellent electrochemical performance and high impact tolerance.

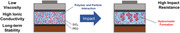

## 1 Introduction

Shear thickening electrolytes (STEs) are promising materials that provide protection to lithium‐ion batteries (LIBs) when exposed to mechanical abuse, thus avoiding catastrophic consequences (e.g., fire, explosion).^[^ ^1^ ^]^ STEs are produced by adding colloidal fillers into a liquid electrolyte. They are composed of colloidal particles dispersed within the liquid electrolyte, having a similar composition as for soggy sand electrolytes.^[^ ^2^ ^]^ Nevertheless, there exists difference between these two electrolytes. STEs possess a unique rheological behavior known as shear thickening effect, that is, a significant and transient increase in viscosity with the increase in shear rate. This behavior arises from shear‐induced particle interaction forming hydroclusters in the electrolyte. Upon impact, the induced shear thickening effect, that is, transformation from liquid state to semi‐solid or solid‐like form, provides the protection.^[^ ^3^ ^]^ Silica nanoparticles are the mostly used fillers and the hydrogen bonding between their silanol groups facilitates the formation of hydroclusters.^[^ ^4^ ^]^ To achieve shear thickening effect, a sufficient amount of silica is needed to allow for adequate interaction between particles forming hydroclusters. The reported usage of silica nanoparticles is in the range of 9 to 30 wt%.^[^ ^5^ ^]^ The introduction of such amounts of fillers into the electrolyte inevitably results in high viscosity, which brings drawbacks of low conductivity and difficulty of injecting into the cell. Fillers with large aspect ratio (e.g., silica nanorods^[^ ^6^ ^]^ and glass fibers^[^ ^7^ ^]^) had been reported to regulate the interparticle attractions and hydrodynamic forces to generate shear thickening effect at low content. However, the viscosity was still high, for example, the STE containing 14.6 vol% of silica nanorods had a high initial viscosity of about 10^3^ Pa·s.^[^ ^6^ ^]^ These high filler content materials also present a high mass density which decreases the energy density of batteries. In addition, they also suffer from a lack of stability, as silica nanoparticles are prone to aggregate and sediment over time in commonly used carbonate‐based liquid electrolytes for LIBs.^[^ ^8^ ^]^ Therefore, an efficient STE with low viscosity, high conductivity and excellent stability is highly desirable for development of impact resistant LIBs.

Low viscosity can be readily realized with low filler content, however, this limits hydrocluster formation as required to produce a shear thickening effect.^[^ ^9^ ^]^ Shear thickening effect can be regulated by introducing additives (e.g., polymer, carbon nanotubes, graphene nanoplates),^[^ ^10^ ^]^ to systems wherein particles are effectively interconnected by them to promote the formation of hydroclusters. Poly(ethylene oxide) (PEO) is a widely used polymer additive that interacts with silica in aqueous‐based shear thickening fluids.^[^ ^11^ ^]^ The shear thickening effect is facilitated by the strong PEO interaction with silica to form a polymer bridged silica network.^[^ ^11a^ ^]^ For example, an aqueous colloidal suspension containing 0.5 wt% PEO and 5 vol% silica exhibited shear thickening,^[^ ^12^ ^]^ while shear thinning was observed for silica only fillers even at a high content up to 55 vol%.^[^ ^13^ ^]^ The adsorption of PEO on the surface of silica also generates a steric repulsion that prevents aggregation and sedimentation.^[^ ^14^ ^]^ In addition, PEO is a widely used polymer host for solid polymer electrolytes used in LIBs given the promotion of ion transport by virtue of the ether oxygen groups' ability to complex with the charge carrier Li^+^.^[^ ^15^ ^]^ These factors combined inspire us to utilize the interaction between polymer chains of PEO and silica nanoparticles to promote the shear thickening effect and to realize a highly conducting, processable electrolyte.

In this work, a new polymer bridged STE is developed by introducing a small amount of PEO interacting with silica as fillers in a commonly used electrolyte (lithium bis(trifluoromethanesulfonyl)imide (LiTFSi) in ethylene carbonate (EC)/dimethyl carbonate (DMC)). The shear thickening behavior can be induced at a very low filler content, as low as 2.2 wt%. The shear thickening magnitude generated with 4.2 wt% filler is ≈5, comparable to the reported STE containing a much higher amount of fillers of over 22 vol%.^[^ ^5^, ^6^, ^7^ ^]^ This electrolyte has excellent long‐term stability, as evidenced by the rheological behavior and ionic conductivity determined over 30 days. The assembled LiFePO4 (LFP)‐graphite cells afforded an electrochemical performance comparable to or better than those that utilize a liquid electrolyte.

## 2 Results and Discussion

**Figure 1:**
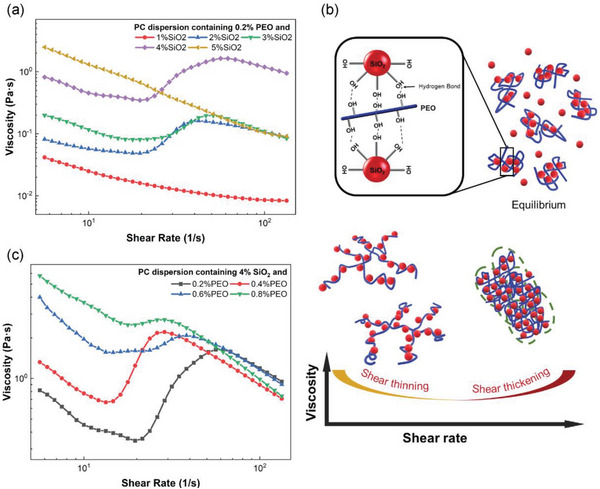
Rheological properties of dispersions. a) Rheological curves of dispersions containing 0.2 wt% of PEO and different amount of fumed silica; b) Schematic illustration of microstructure evolution under shear rates for different rheological behaviors; c) Rheological curves of dispersions containing 4 wt% fumed silica with different contents of PEO.

Rheological studies were conducted on the dispersion containing various PEO content (0.1 to 0.8 wt%) and fumed silica (1 to 5 wt%) in propylene carbonate (PC). The PC solvent and the solution containing PEO alone displayed Newtonian behavior with a constant viscosity throughout the entire range of shear rates investigated (Figure S1, Supporting Information). The addition of increasing amount of PEO resulted in a corresponding increase in viscosity. Fumed silica was composed of primary aggregated spherical nanoparticles in a branched chain‐like structure (Figure S2, Supporting Information). After adding silica nanoparticles, the rheological behavior indicated non‐Newtonian behavior (**Figure** **1**a, Figures S3–S5, Supporting Information), a shear‐dependent viscosity. Dispersions with no less than 0.2 wt% PEO when coupled with lower than 2 wt% or higher than 5 wt% silica underwent a shear thinning response over the applied shear stress range. This may be explained by the fact that insufficient particles could be connected to form hydroclusters at less than 2 wt% silica. When the silica content reached 5 wt%, polymer chains may be blocked by excessive silica thus diminishing the polymer bridging function.^[^ ^16^ ^]^ Within the range of 2–4 wt% silica, the flow curves displayed three distinct regimes: shear thinning at low shear rates, shear thickening after a critical shear rate, and shear thinning at very high shear rates (Figure 1a). Such shear thickening behaviors are attributed to the interaction between entangled PEO chains and silica particles, similar to the shear thickening fluids containing silica only.^[^ ^14a^ ^]^ It should be noted that the dispersion containing 0.1 wt% of PEO and 4 wt% of silica still displayed shear thinning behavior and also sedimentation was observed over 24 h indicating instability (Figure S5, Supporting Information). Therefore, the dispersion composed with 0.1 wt% of PEO will not be further investigated. All the dispersions with shear thickening behaviors, that is, containing fumed silica (2–4 wt%) and PEO (0.2–0.8 wt%), were homogenous (Figure S6, Supporting Information) and stable over 30 days.

The interpretation of this phenomenon is illustrated in Figure 1b. In the suspension, polymer is adsorbed onto silica particles via hydrogen bonding between the ‐OH groups on the PEO chain and the silanol groups (hydroxyl functional groups) on fumed silica.^[^ ^17^ ^]^ Polymer chains are in their naturally coiled state when adsorbed onto silica nanoparticles.^[^ ^10a^ ^]^ Under low shear rates, these polymer coils are extended and the electrostatic interactions^[^ ^17^ ^]^ are reduced, thus the suspension became anisotropic resulting in increased fluidity, that is, decreased viscosity. The repulsion between “particles,” for example, PEO and PEO, silica particles and silica particles, PEO and silica particles, dominates at low shear rates. Beyond a critical shear rate, this repulsion is broken and hydrodynamic forces overcome the repulsive force between “particles,” enabling extended polymer chains to bridge other silica particles leading to the formation of large 3D interconnected aggregates (i.e., shear‐induced clusters).^[^ ^18^ ^]^ These hydroclusters impede fluid flow resulting in a significant increase in viscosity. These large interconnected aggregates were broken down under even higher shear stress, and the strong shear flow forced the clusters to separate resulting in decreased viscosity.^[^ ^14^, ^19^ ^]^

In this work, dispersions containing 0.2 wt% of PEO were selected to investigate the role and function of PEO in promoting shear thickening. Shear thickening effects increased in magnitude with increasing silica content from 2 to 4 wt%, accompanied by concomitant decrease in critical shear rate. This may be ascribed to larger and stronger hydroclusters formed due to the increased number of silica particles adsorbing onto polymer chains. The suspension composed of 4 wt% SiO_2_ and 0.2 wt% PEO presented the largest shear thickening magnitude with the highest peak shear viscosity of 1.68 Pa·s at a shear rate of 61.9 1/s. This was approximately five times higher than the critical shear viscosity of 0.34 Pa·s at 19.6 1/s, which was comparable to the reported STE containing very high content of fillers such as 30 wt% silica particles grafted with PMMA brushes,^[^ ^5c^ ^]^ 22.6 vol% silica nanorods,^[^ ^6^ ^]^ and 42.2 vol% glass fibers.^[^ ^7^ ^]^ It is worth noting that in our work the shear thickening behavior could be generated with only 2.2 wt% fillers, the lowest content reported to date. Due to the low content of fillers, this STE displayed a low initial viscosity of ≈3.2 Pa·s with 2.2 wt% filler and ≈ 86.3 Pa·s with 4.2 wt% filler (Figure S7, Supporting Information), respectively. The viscosity was increased to 158.6 Pa·s with 9.1 wt% silica, 296.5 Pa·s with 15 wt% silica, and 37 450 Pa·s with 20 wt% silica (Table S1, Supporting Information). This low viscosity electrolyte may bring significant benefit to the battery assembly, as it can be easily injected into the cell like a liquid electrolyte. This low viscosity STE also exhibited good wetting behavior comparable to liquid electrolyte, which is beneficial to reduce the internal resistance for high cell performance (Figure S8, Supporting Information).

The effect of polymer content (0.2 – 0.8 wt%) on the shear thickening behavior of dispersions containing 4% silica was investigated (Figure 1c). The shear thickening magnitude decreased with increasing PEO loading. The shear thickening effect was negligible when the PEO content reached 0.8 wt%. This may be attributed to excess PEO blocking some adsorption sites for bridging silica particles to form hydroclusters.^[^ ^16a^ ^]^ The increased viscosity with filler content (total amount of PEO and SiO_2_) can be ascribed to physical interactions between the components. In this system, the largest shear thickening magnitude occurred in the dispersion containing 0.2 wt% of PEO and 4 wt% of silica.

This dispersion with a low filler content of 4.2 wt% displayed a low mass density, which was just slightly higher than pure PC solvent by 3% (Table S1, Supporting Information). The dispersion with a higher filler amount of 15 wt% silica displayed a much higher mass density, 21% more than PC. When the filler content of silica reached 20 wt%, the mass density became 39% higher. It can be deduced that much higher mass density is expected with even higher amounts of fillers. This also verifies the realization of a lightweight STE via the polymer bridging strategy in this work, which is highly desirable for fabricating lightweight batteries.

Using rheology, the stability of dispersions containing 0.2% PEO and 4% SiO_2_ was investigated over 30 days. The rheological curve at day 30 demonstrated a similar viscosity with a slightly stronger shear thickening effect (Figure S9, Supporting Information), and this verified the stability of this shear thickening suspension. The ability to retain shear thickening behavior with no significant viscosity variation during a period is a widely accepted measure of stability of a shear thickening fluid.^[^ ^20^ ^]^ This long‐term stability may be attributed to PEO being effective at governing interaction between particles and preventing sedimentation.^[^ ^21^ ^]^ In contrast, the viscosity of a dispersion with 15 wt% SiO_2_ increased from 296.5 to 75 476 Pa·s over 30 days (Figure S10, Supporting Information).

### 2.1 Electrochemical Performance

**Figure 2:**
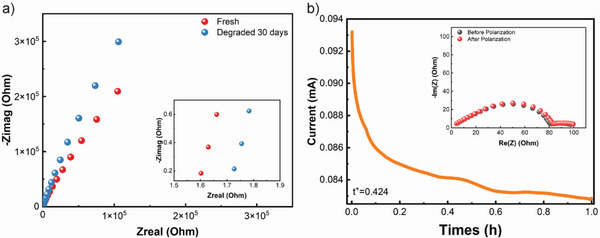
Electrochemical properties of STE: a) Electrochemical impedance spectra (EIS) of a stainless steel (SS)/SS symmetric cell with STE at room temperature on Day 1 and Day 30, inset shows the enlarged impedance data of the highlighted area; b) Chronoamperometry of a Li/Li symmetric cell with STE during polarization at an applied voltage of 10 mV, inset shows the impedance spectra before and after polarization.

Electrochemical performance tests were conducted in coin cells. LiTFSi was used in this work due to its resistance toward hydrolysis, wide electrochemical window and thermal stability.^[^ ^22^ ^]^ The ionic conductivity of STE (i.e., 1.0 m LiTFSi in EC/DMC (1:1) electrolyte containing 4% fumed silica and 0.2% PEO) was characterized at room temperature using AC impedance (**Figure** **2**a). A typical Nyquist plot was presented consisting of a semicircle at high frequency and a sloping line at low frequency. The bulk resistance is defined as the sum of internal resistance from electrode, electrolyte, and separator.^[^ ^23^ ^]^ This was found to be 1.6 Ω, slightly lower than 2.1 Ω for liquid electrolyte with no fillers (Figure S11, Supporting Information). The estimated ionic conductivity of the STE was ≈5.1 mS cm^−1^ (Equation S1, Supporting Information), higher than the 4.0 mS cm^−1^ observed for liquid electrolyte. This higher ionic conductivity is due to the well‐dispersed fillers (silica particles and PEO) in the electrolyte acting as Li^+^ carriers forming an enhanced pathway for Li^+^ ion conduction to promote the motion of ionic charge.^[^ ^24^ ^]^ A high lithium ion transference number (t^+^) is needed for effective ion transport and uniform Li deposition.^[^ ^25^ ^]^ A symmetrical Li metal/electrolyte/Li metal coin cell was used to determine t^+^ (Equation S2, Supporting Information) via EIS coupled with potential polarization technique as reported.^[^ ^26^ ^]^ This STE displayed a t^+^ of 0.42 (Figure 2b), higher than the 0.33 for liquid electrolyte (t^+^, Figure S12, Supporting Information). The increased transference number, that is, more cations contributing to the total electric current, can be ascribed to the hydrogen bond interaction between the silica surface and PEO chain in the liquid electrolyte, benefiting the dissociation of Li^+^TFSi^−^ ion pairs and increasing the concentration of Li+ ions.^[^ ^27^ ^]^ Furthermore, the stability of this electrolyte was investigated in terms of ionic conductivity over a timeframe of 30 days. No apparent difference was observed in the Nyquist plots between Day 1 and Day 30 (Figure 2a). The ionic conductivity became slightly lower, 4.7 mS cm^−1^ versus 5.1 mS cm^−1^. This result strongly verifies the excellent stability of this STE, in agreement with the rheological properties over time (Figure S9, Supporting Information). To date, the reported ionic conductivity stability of STE was for PMMA‐grafted silica particles filler over a much shorter timeframe of 24 hrs.^[^ ^5c^ ^]^

**Figure 3:**
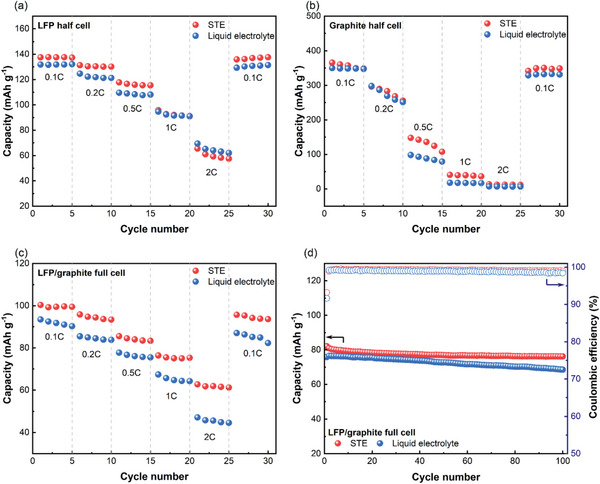
Electrochemical performance of LFP/Li half‐cells, graphite/Li half‐cells, and LFP‐graphite full‐cells with STE and liquid electrolyte: a–c) Rate performance; d) Cycling performance of LFP/graphite full‐cells at 0.5C.

Linear sweep voltammetry was performed to test the electrochemical stability of the STE (Figure S13, Supporting Information), and the electrochemical stability window of 5.4 V was comparable to that observed with liquid electrolyte (5.6 V), certifying that this STE is capable of being used in high voltage LIBs.^[^ ^28^ ^]^ The application of STE in LIBs was investigated in half‐cells and full cells. Half‐cells were fabricated with Li metal foil electrodes coupled with lithium iron phosphate (LFP) or graphite electrodes. These cells were activated at 0.05 C for three cycles and then consecutively cycled for 5 cycles at current rates of 0.1 C, 0.2 C, 0.5 C, 1 C, and 2 C, then back to 0.1 C, followed by the cycling stability tests for 100 cycles at 0.5 C. Half cells with STE demonstrated a higher initial discharge capacity and an improved rate performance compared to the cells with liquid electrolyte (**Figure** **3**a,b, Figure S14, Supporting Information). The LFP half cells with STE showed a typical discharge plateau of ≈3.40 V, similar to that with liquid electrolyte (Figure S14, Supporting Information). When the current was reverted to 0.1 C, the initial capacities were nearly fully recovered, evidencing the excellent reversibility.

The LFP/graphite full cell with STE exhibited higher capacities at all the applied current densities and comparable cycling stability to those with liquid electrolyte (Figure 3c,d). They were 100 mAh g^−1^ versus 93 mAh g^−1^, 96 mAh g^−1^ versus 85 mAh g^−1^, 86 mAh g^−1^ versus 77 mAh g^−1^, 76 mAh g^−1^ versus 67 mAh g^−1^, and 63 mAh g^−1^ versus 47 mAh g^−1^ at 0.1 C, 0.2 C, 0.5 C, 1C, and 2C, respectively. They also showed a good capacity retention rate of 93% after 100 cycles and a high coulombic efficiency (CE) of 98.9%. To investigate the electrochemical behavior changes over cycling, we further performed the differential capacity analysis for 1^st^, 2^nd^, 10^th^, and 20^th^ cycle (Figure S15, Supporting Information). The cell with STE displayed comparable degradation trend to liquid electrolyte, verifying the stability of fillers. These excellent electrochemical properties in both half‐cells and full cells verify the good compatibility of STE with both LFP and graphite electrodes, which shows promise for applications in LIBs.

### 2.2 Impact Test

**Figure 4:**
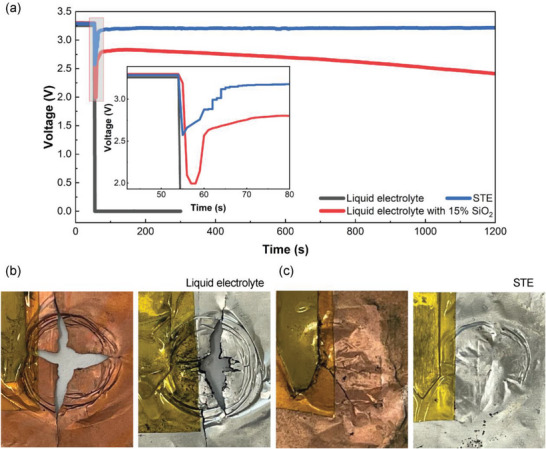
The impact response of Li‐ion pouch cells with different electrolytes of liquid electrolyte, STE, and liquid electrolyte containing 15% SiO_2_ under an impact energy of 2.06 J: a) Voltage response during the period of 1200s, inset shows the enlarged view of highlighted area; b,c) Electrodes after impacts on cells (anode was the side facing the dropping ball).

To evaluate the ability to mitigate against mechanical abuse, impact tests were performed on LFP/graphite pouch cells with a structure containing a liquid electrolyte as control. The experimental setup followed the reported procedure (Figure S16, Supporting Information).^[^ ^5^, ^7^ ^]^ A stainless‐steel ball with a diameter of 35 mm was released from a height of 120 cm through a PVC pipe to punch the cell. The applied impact energy was calculated based on the weight of the ball and drop height (Equation S3, Supporting Information). It was 2.06 J in this work, which was slightly higher than that 2.04 J.^[^ ^7^ ^]^ The open circuit voltage (OCV) was monitored during the impact test. The cell with STE showed a slight voltage drop of ≈20% at the beginning of the impact, but it quickly recovered to nearly the initial voltage (**Figure** **4**a), a similar phenomenon reported for the cell with STE containing 37.5 vol% glass fibers.^[^ ^7^ ^]^ In contrast, the voltage of the cell with liquid electrolyte decreased sharply to nearly 0 V, reflecting a catastrophic internal short circuit. This result verifies the protection function of the STE, which can withstand mechanical impact to prevent the occurrence of a short circuit associated with catastrophic fire or explosion. The pouch cells were disassembled for further examination. For the cell with liquid electrolyte, both cathode and anode were broken, indicative of a high risk of catastrophic consequences upon impact (Figure 4b). In contrast, only two fine cracks were found on the anode which directly received the strike (facing the ball). No cracks, but only a dent was identified on the cathode (Figure 4c). These observations provide clear evidence of the protection provided by the STE against mechanical impact.

To further highlight the performance of the novel STE introduced here and rule out the protection from electrolyte with high viscosity, a more viscous electrolyte (with filler of 15% SiO_2_) than STE but without bridging effect was used to assemble the cell. It displayed a nearly 3.5 times higher viscosity than that containing 4.2 wt% fillers, however, it displayed shear thinning effect over the same shear rate range (Figure S10, Supporting Information). Upon impact, this cell exhibited a larger voltage drop (≈40%) that bounced back to ≈85% of its original voltage in 20 s (Figure 4a). However, this recovered voltage underwent a gradual decline to near 0 V in 6000 s (Figure S17, Supporting Information), indicative of the occurrence of an internal short circuit. This result clearly demonstrated that the electrolyte with high viscosity only cannot provide sufficient protection against mechanical abuse. It is the shear thickening effect, formation of hydroclusters, that provided the impact resistance. This impact result also suggested that our polymer bridged STE with only 4.2 wt% of fillers can endure similar high energy impacts as reported STE with 37.5 vol% glass fibers.^[^ ^7^ ^]^

## 3 Conclusion

A stable and efficient shear thickening electrolyte has been developed by introducing PEO to interact with silica nanoparticles as fillers. This polymer bridged STE features low viscosity, high ionic conductivity, excellent long‐term stability and with high impact tolerance – a formidable combination of properties. The formation of hydroclusters for the shear thickening effect is promoted via the interaction between silica particles and polymer chains. The bridging of PEO with silica particles not only prevents sedimentation and aggregation but also improves the ionic conductivity and mobility due to hydrogen bond interactions. The assembled lithium‐ion cells have displayed comparable to or higher electrochemical performance than those with liquid electrolyte, but with high impact resistance. The low viscosity of the STE with the bonus of lightweight is expected to enable the realization of impact resistant and lightweight LIBs. This work provides a simple method for designing STE and also a new solution for safe LIBs.

## 4 Experimental Section

### Materials

Fumed silica nanoparticles (S5505: aggregated particle size, ≈0.2–0.3 µm; surface area, 200 m^2^ g^−1^; density, 36.8 kg m^−3^), poly(ethylene oxide) (PEO, Mw = 400 000), propylene carbonate (PC, 99.7%), ethylene carbonate (EC, 99%), dimethyl carbonate (DMC, ≥ 99%), and bis(trifluoromethane)sulfonimde lithium salt (LiTFSi, 99.95%) were purchased from Sigma‐Aldrich. The LiFePO4 (LFP) cathode and graphite anode were purchased from MTI Corporation.

### Fabrication of PC Carrier Medium

PEO powder in a designated weight was added into PC solvent (3 mL). The obtained solution was heated at 70 °C and magnetically stirred for 3 h. After cooling down to room temperature, the carrier medium was obtained. The content of PEO in PC was 0.1, 0.2, 0.4, 0.6, and 0.8 wt%.

### Fabrication of Shear Thickening Fluids

Shear thickening fluids were fabricated by adding pretreated silica nanoparticles into PC carrier medium. Silica nanoparticles were dried in a vacuum oven at 110 °C for 24 h to remove the absorbed moisture. Then silica nanoparticles were gently added into the carrier medium under magnetic stirring (200 rpm) at room temperature for 2 h to obtain a homogeneous solution. The amount of silica particles added was 1, 2, 3, 4, and 5 wt%.

### Fabrication of Shear Thickening Electrolytes

STEs were prepared by adding silica and PEO into liquid electrolyte, 1 m LiTFSI in EC/DMC (1:1). As the lithium‐ion battery system is very sensitive to moisture, silica and PEO were subjected to a very strict vacuum‐drying process: 168 h at 110 °C for silica particles, and 72 h at 50 °C for PEO. All dried silica nanoparticles and PEO were transferred into a glove box for the fabrication of STE. PEO was first added into the liquid electrolyte and stirred at 200 rpm for 1 h to obtain the carrier medium. Then, the carrier medium was gently added into the vial containing silica nanoparticles, and such suspension was magnetically stirred at 200 rpm for 2 h to obtain a homogeneous solution.

### Rheological Measurement

An AR‐G2 (TA instrument) rheometer was employed to measure rheological properties at a measuring temperature of 25 °C. All the tests were performed using a flat PP‐40 Sand Blast measuring geometry with a 40 mm diameter and a gap size of 0.335 mm. The amount of testing sample was 485 µL. These samples were pre‐sheared at a low shear rate to remove any shear history induced during the sample preparation and handling before conducting the rheological measurements. The pre‐shear conditions were set as a pre‐shear duration of 600 s at a 1 1/s shear rate and equilibration duration of 900 s. In the steady‐state shear flow test, the specimen was isothermally rotated under the applied shear rate from 10^−1^ to 10^3^ 1/s to measure viscosity upon the shear rate variation.

### Material Characterizations

The morphology and structure of fumed silica were characterized using a field‐emission scanning electron microscope (JEOL 7500 FESEM).

### Electrochemical Measurement

Linear sweep voltammetry of electrolytes in a stainless‐steel/electrolyte/Li metal coin cell were characterized by using Solartron SI 1287 electrochemical system. It was scanned from open potential to 6.5 V (versus Li/Li^+^) at a scan rate of 5 mV s^−1^. The ionic conductivity was investigated in the symmetrical stainless‐steel/electrolyte/stainless‐steel coin cell using VSP potentiostat (Bio‐logic) and EC‐lab software (11.21).

For lithium‐ion transference number measurement, symmetric Li/electrolyte/Li cells were tested via VSP potentiostat (Bio‐logic). Currents at the initial and steady states were measured when a polarization voltage of 10 mV was applied.

### Fabrication of Battery Cells

LFP and graphite electrodes were dried in a vacuum oven at 120 °C overnight and transferred into a glove box for use. In a LFP or graphite half‐cell, lithium foil was the coupled electrode. In full‐cells, LFP and graphite electrodes were used as cathode and anode, respectively. They were assembled into coin cells for battery performance test. Pouch cells (LFP/graphite full‐cell) were assembled for the impact test. Galvanostatic charge–discharge properties were performed on a LAND CT2001A battery test system (Wuhan Jinnuo Electronics Co.Ltd.).

### Impact Test on Pouch Cells

The experimental setup is shown in Figure S16, Supporting Information. A 35 mm stainless‐steel ball (175 g) was used to hit the pouch cell to induce an energy impact of 2.06 J. The ball was vertically positioned 120 cm above the target and dropped via a PVC pipe with a diameter of 40 mm onto the pouch cell. The open circuit voltage was monitored by a LAND CT2001A battery test system (Wuhan Jinnuo Electronics Co.Ltd).

## Conflict of Interest

The authors declare no conflict of interest.

## Supporting information

Supporting Information

## Data Availability

The data that support the findings of this study are available from the corresponding author upon reasonable request.
