# Supplementary material for: Polyethylene Oxide (PEO) Provides Bridges to Silica Nanoparticles to Form a Shear Thickening Electrolyte for High Performance Impact Resistant Lithium‐ion Batteries
Source: Adv Sci (Weinh). 2023 Aug 6;10(28):2302844. doi: 10.1002/advs.202302844 (PMC10558684; doi:10.1002/advs.202302844)
Supplement: Supplementary file 1 — Supporting Information [file ADVS-10-2302844-s001.pdf]

ADVANCED SCIENCE
Open Access

Supporting Information

for *Adv. Sci.*, DOI 10.1002/advs.202302844

Polyethylene Oxide (PEO) Provides Bridges to Silica Nanoparticles to Form a Shear Thickening Electrolyte for High Performance Impact Resistant Lithium-ion Batteries

Zhiqi Chen, Yunfeng Chao, Sepidar Sayyar, Tongfei Tian, Kezhong Wang, Yeqing Xu, Gordon Wallace, Jie Ding* and Caiyun Wang**

ADVANCED SCIENCE
Open Access

Supporting Information

for *Adv. Sci.*, DOI 10.1002/advs.202302844

Polyethylene Oxide (PEO) Provides Bridges to Silica Nanoparticles to Form a Shear Thickening Electrolyte for High Performance Impact Resistant Lithium-ion Batteries

Zhiqi Chen, Yunfeng Chao, Sepidar Sayyar, Tongfei Tian, Kezhong Wang, Yeqing Xu, Gordon Wallace, Jie Ding* and Caiyun Wang**

Supporting Information

Polyethylene Oxide (PEO) Provides Bridges to Silica Nanoparticles to Form A Shear Thickening Electrolyte for High Performance Impact Resistant Lithium-ion Batteries

Zhiqi Chen[1], Yunfeng Chao[1], Sepidar Sayyar[1, 2,] Tongfei Tian[3], Kezhong Wang[1], Yeqing Xu[1], and Gordon Wallace[1,2], Jie Ding[4*], Caiyun Wang[1*]*

1. ARC Centre of Excellence for Electromaterials Science, Intelligent Polymer Research Institute, AIIM Facility, Innovation Campus, University of Wollongong, NSW 2500, Australia
2. Australian National Fabrication Facility – Materials Node, Innovation Campus, University of Wollongong, Wollongong, NSW 2500, Australia
3. School of Science, Technology and Engineering, University of the Sunshine Coast, Sippy Downs, QLD 4556, Australia
4. Platforms Division, Defence Science & Technology Group, 506 Lorimer Street, Fishermans Bend, VIC, 3207 Australia

E-mail: caiyun@uow.edu.au; jie.ding@defence.gov.au; gwallace@uow.edu.au.

Supplementary Figures:

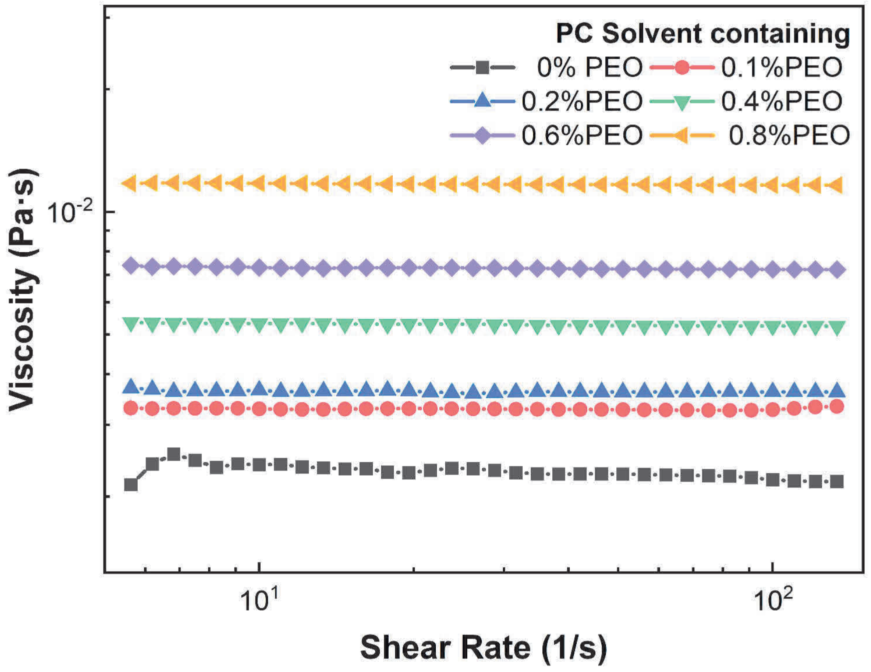

Figure S1. Rheological curves of PC solvent containing different contents of PEO: 0, 0.1 wt%, 0.2 wt%, 0.4 wt%, 0.6 wt%, and 0.8 wt%.

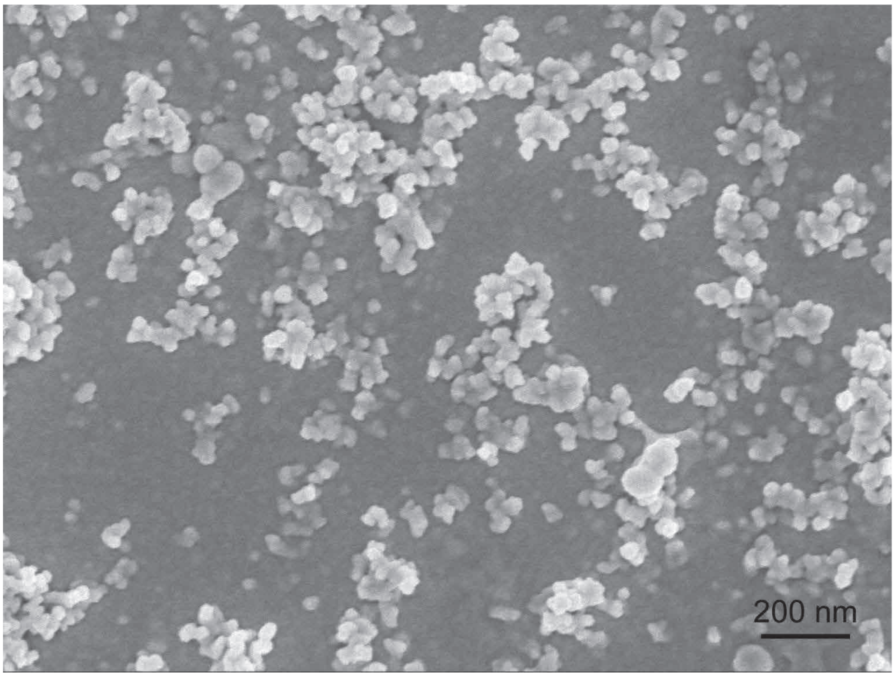

Figure S2. SEM images of fumed silica.

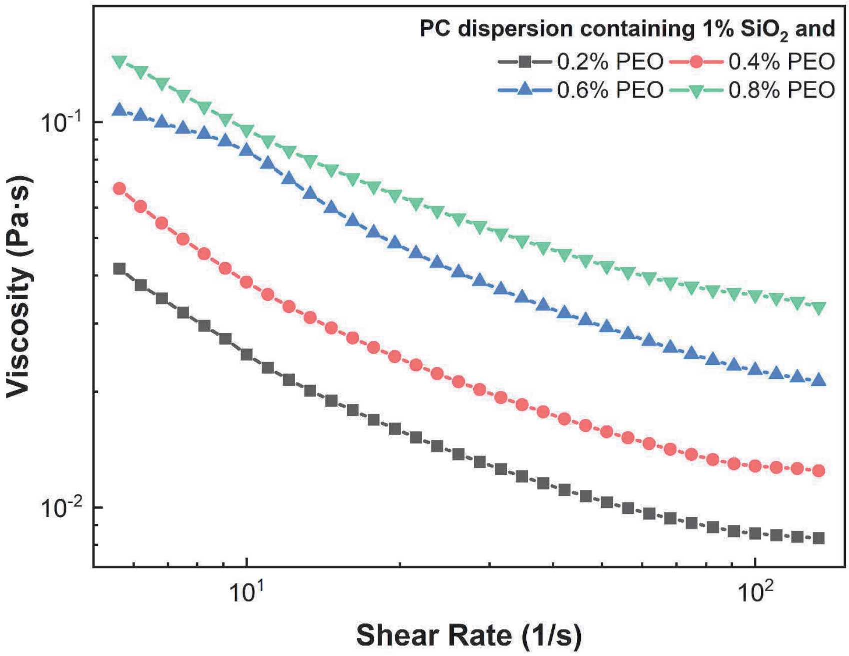

Figure S3. Rheological curves of PC dispersion containing 1 wt% silica with different contents of PEO: 0.2 wt%, 0.4 wt%, 0.6 wt%, and 0.8 wt%.

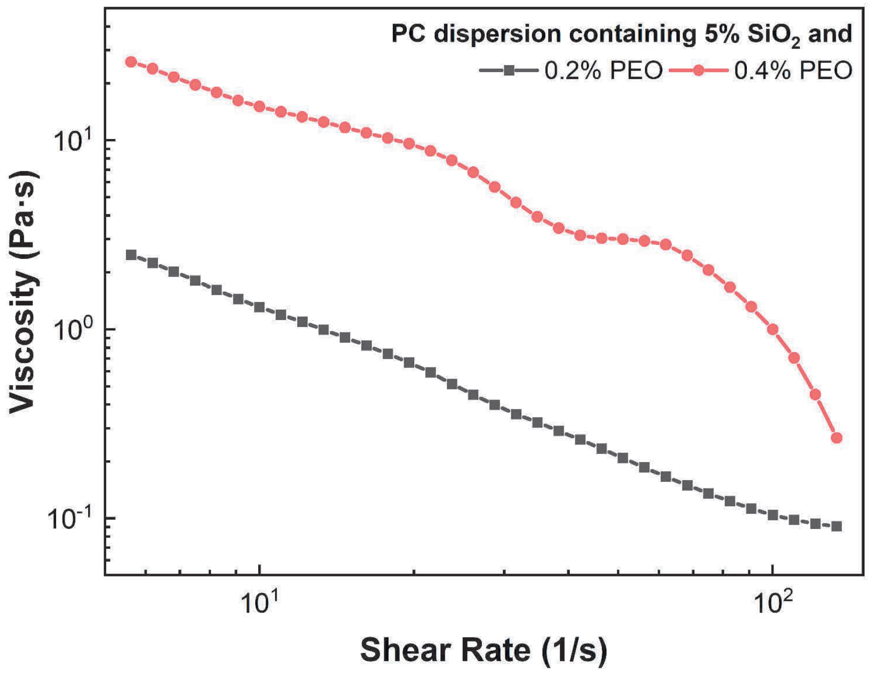

Figure S4. Rheological graph of PC dispersion containing 5 wt% silica and different contents of PEO: 0.2 wt% and 0.4 wt%.

(a)

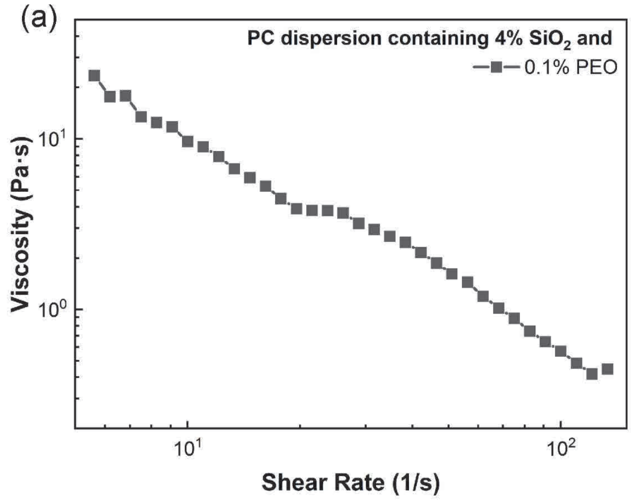

(b)

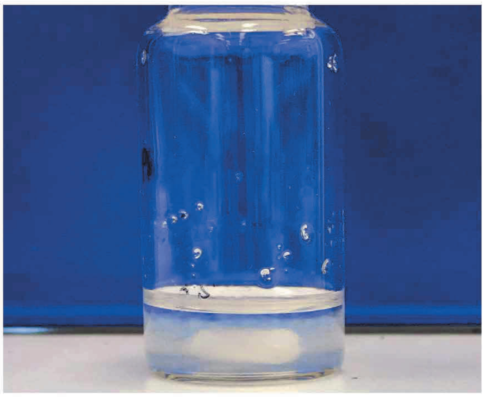

Figure S5. PC dispersion containing 0.1 wt% PEO and 4 wt% silica: (a) Rheological curve; (b) Photo of the dispersion over 24 hrs.

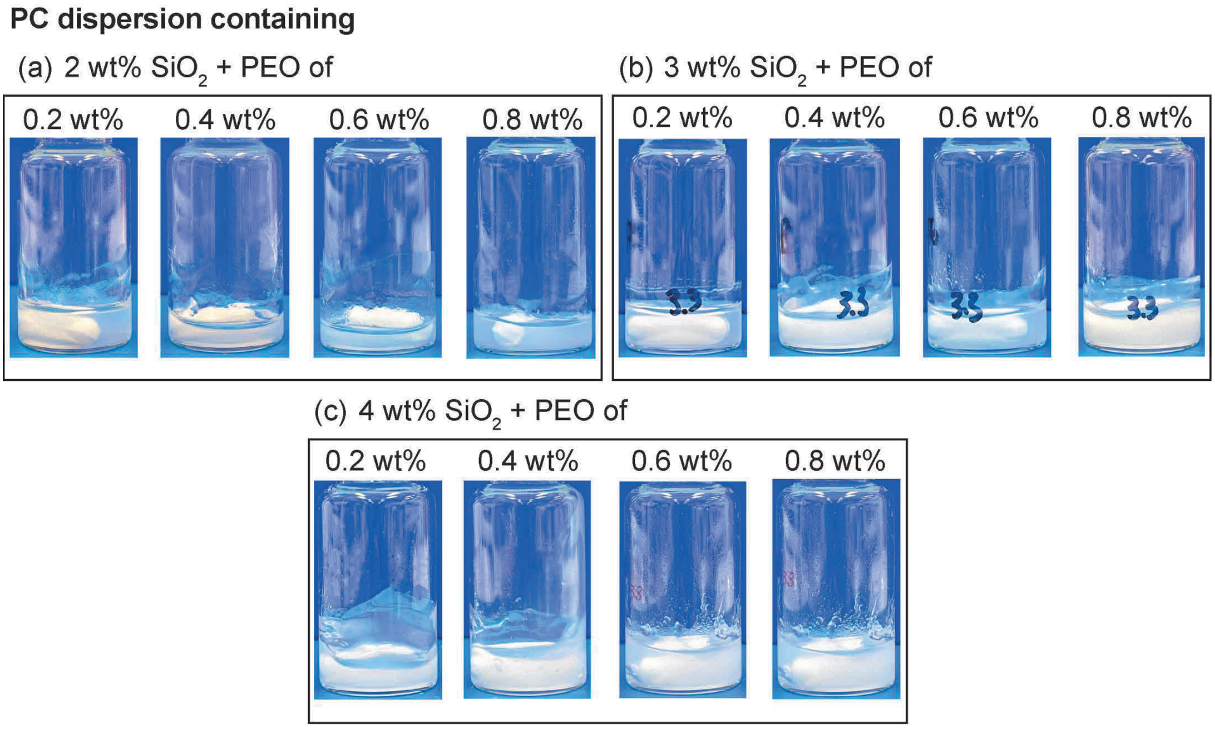

Figure S6. Photos of PC solvent contain different contents of PEO and silica particles after 24 hours' placement (a-c): 2 wt% (a), 3 wt% (b), and 4 wt% (c) of SiO_2 in the carrier medium with 0.2%, 0.4%, 0.6%, and 0.8% PEO.

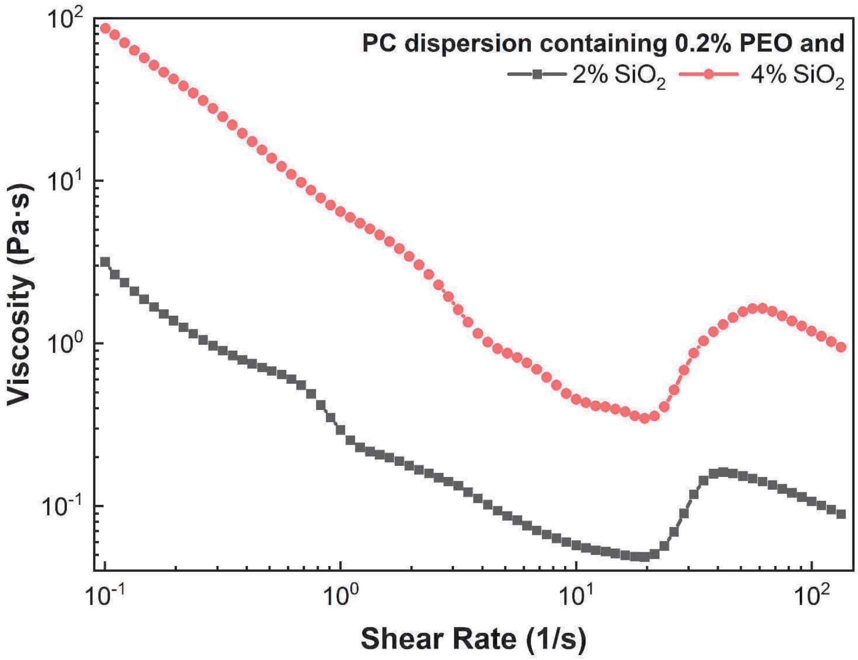

Figure S7. Rheological graph of PC dispersion containing 0.2 wt% PEO and different contents of silica: 2 wt% and 4 wt% during the shear rate from 0.1 to 150 1/s.

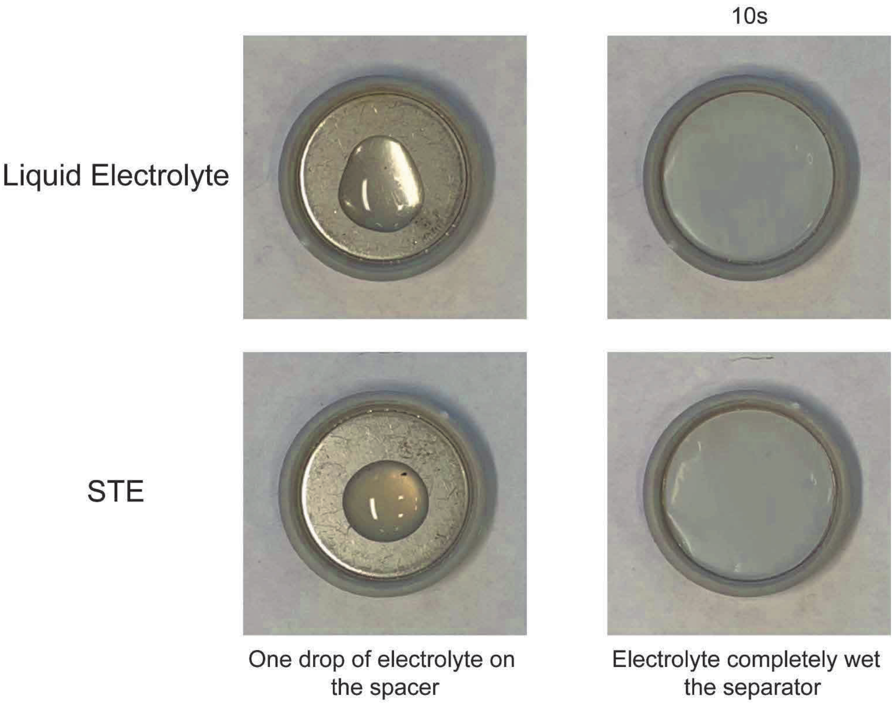

Figure S8. Wetting behaviour of electrolytes with separator: liquid electrolyte and STE.

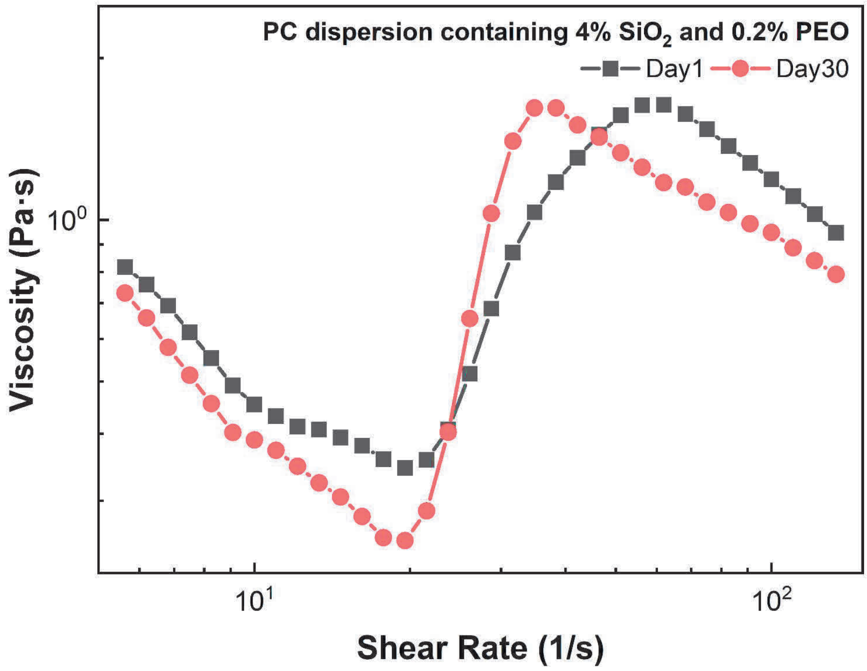

Figure S9. Stability comparison of viscosity as function of shear rates for PC dispersions containing 4 wt% SiO_2 and 0.2 wt% PEO at Day 1 and Day 30.

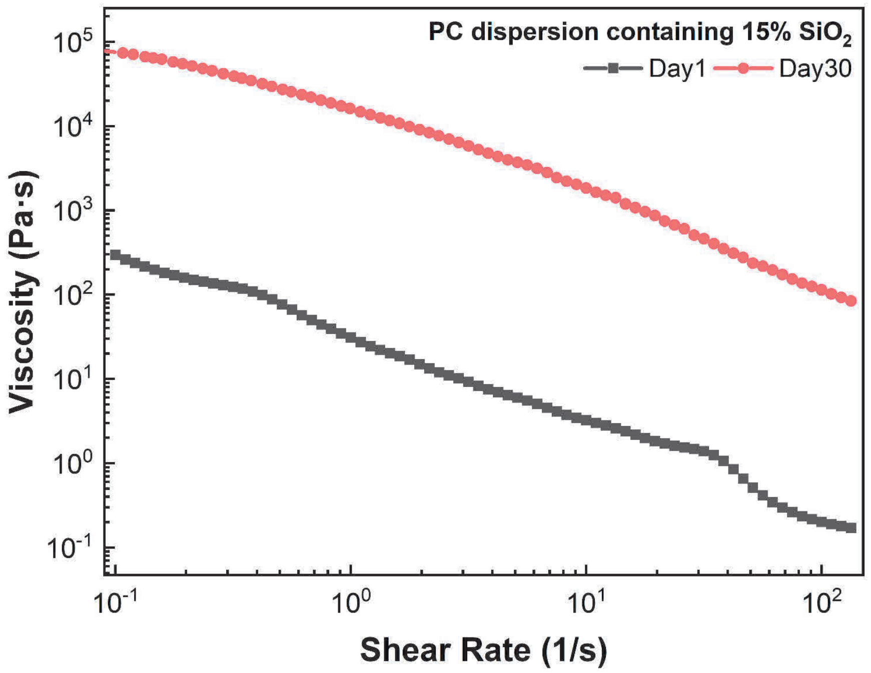

Figure S10. Stability comparison of viscosity as function of shear rates for PC dispersions containing 15 wt% SiO_2 at Day 1 and Day 30.

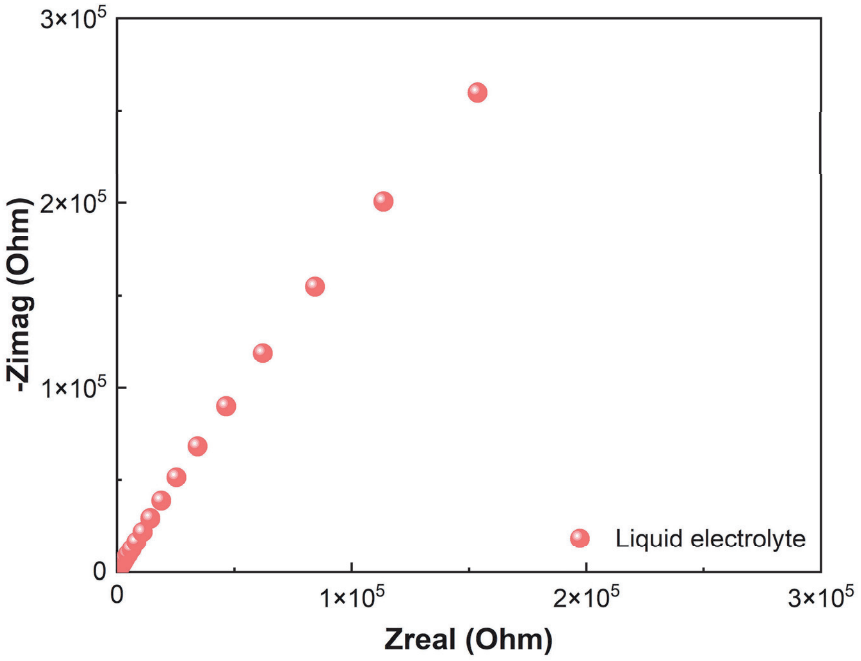

Figure S11. Electrochemical impedance spectra (EIS) of liquid electrolyte at room temperature.

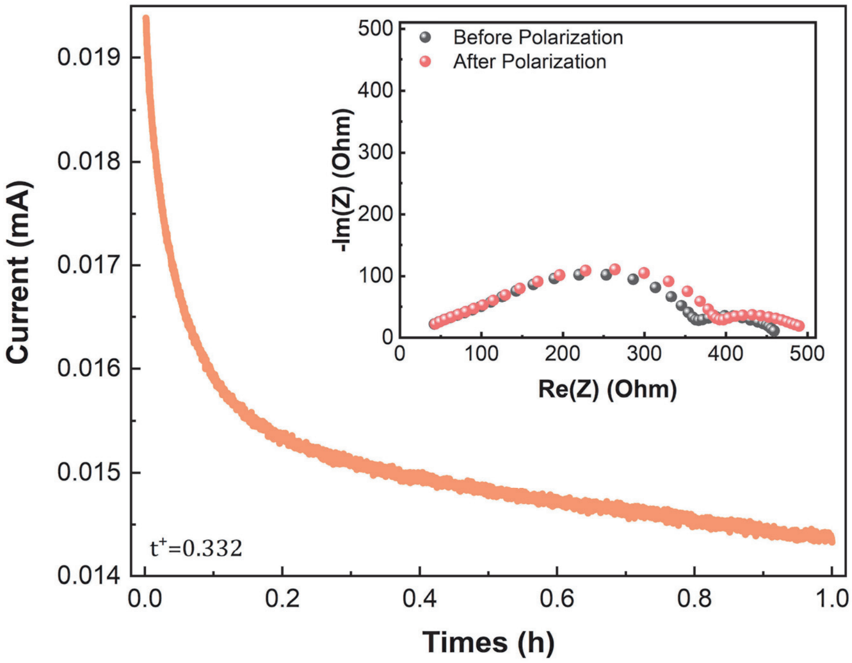

Figure S12. Chronoamperometry of the Li/Li symmetric cell for liquid electrolyte during polarization at an applied voltage of 10 mV, inset shows the EIS before and after polarization.

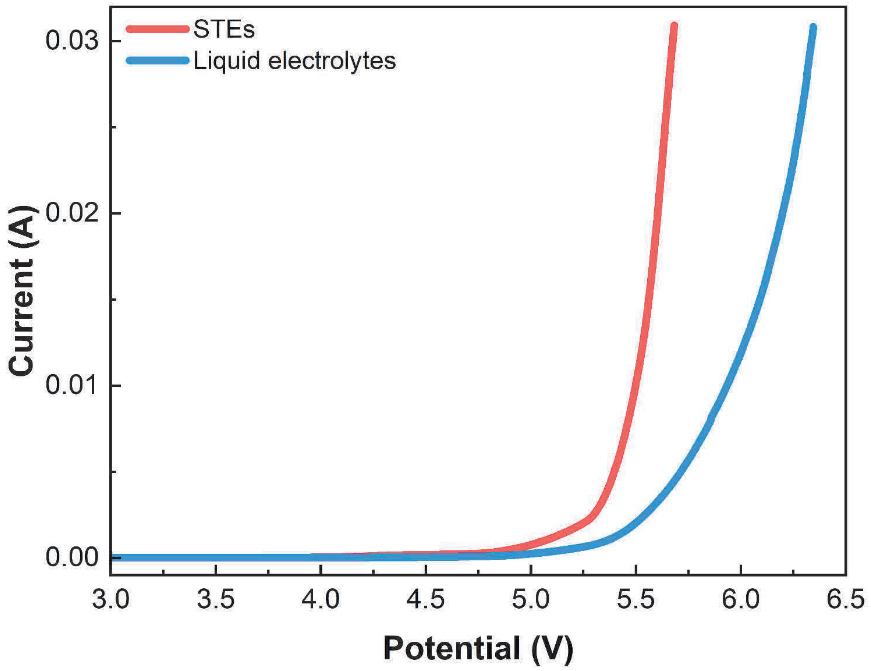

Figure S13. Linear sweep voltammograms of a stainless-steel (SS)/lithium foil (Li) cell with STE and liquid electrolyte swept from open circuit potential to 6.5 V at a scan rate of 5 mVs^{-1}.

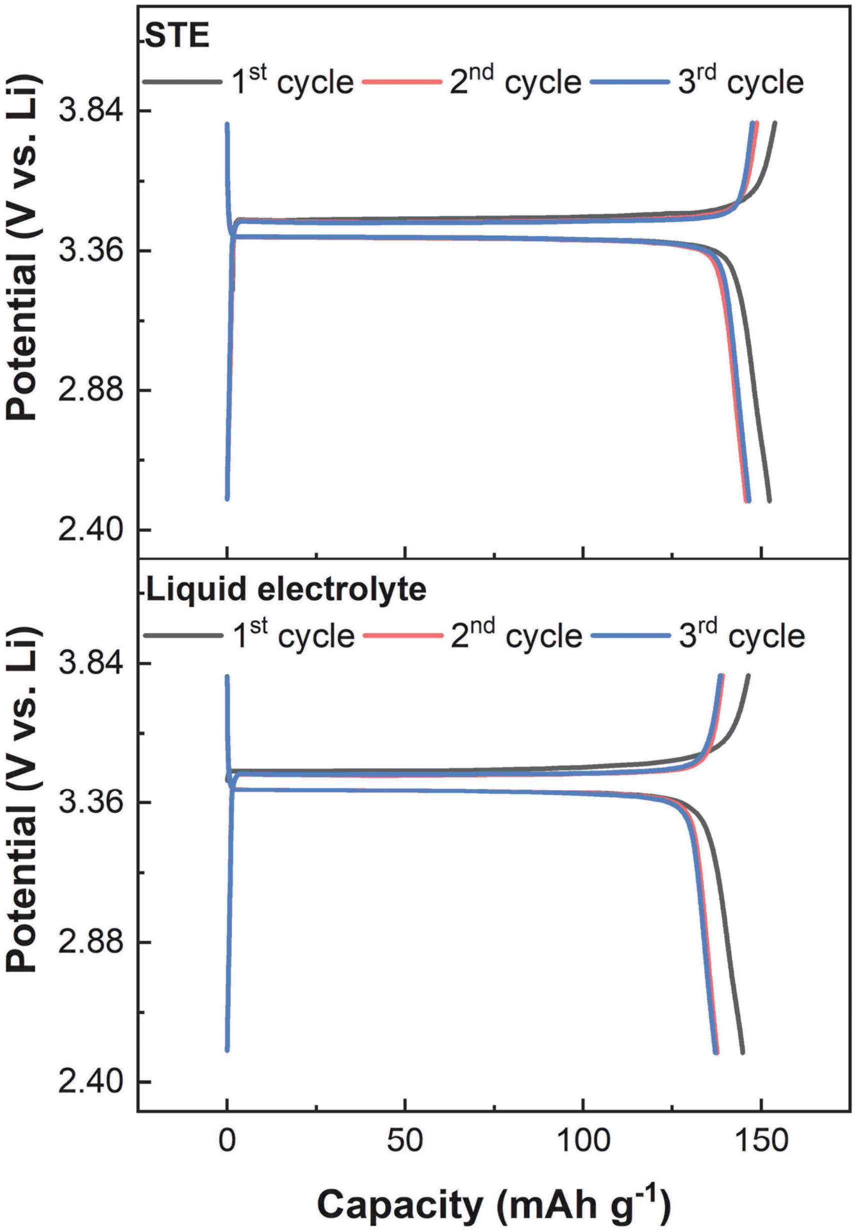

Figure S14. Charge and discharge profiles for LFP half-cell with STE and liquid electrolyte at a 0.05 C for the first three cycles.

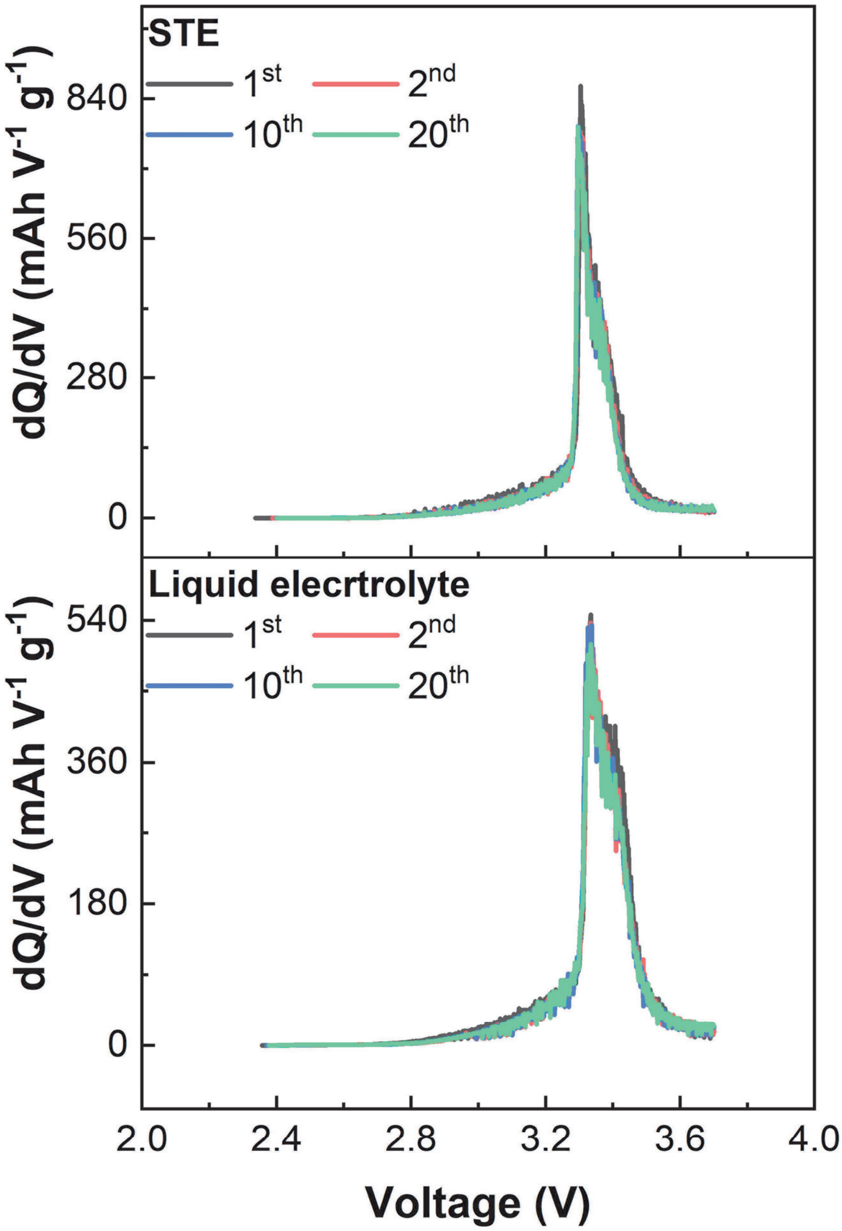

Figure S15. Incremental capacity curves of LFP-graphite cycled cell with STE and liquid electrolyte at a 0.5 C for 1st, 2nd, 10th, and 20th cycle.

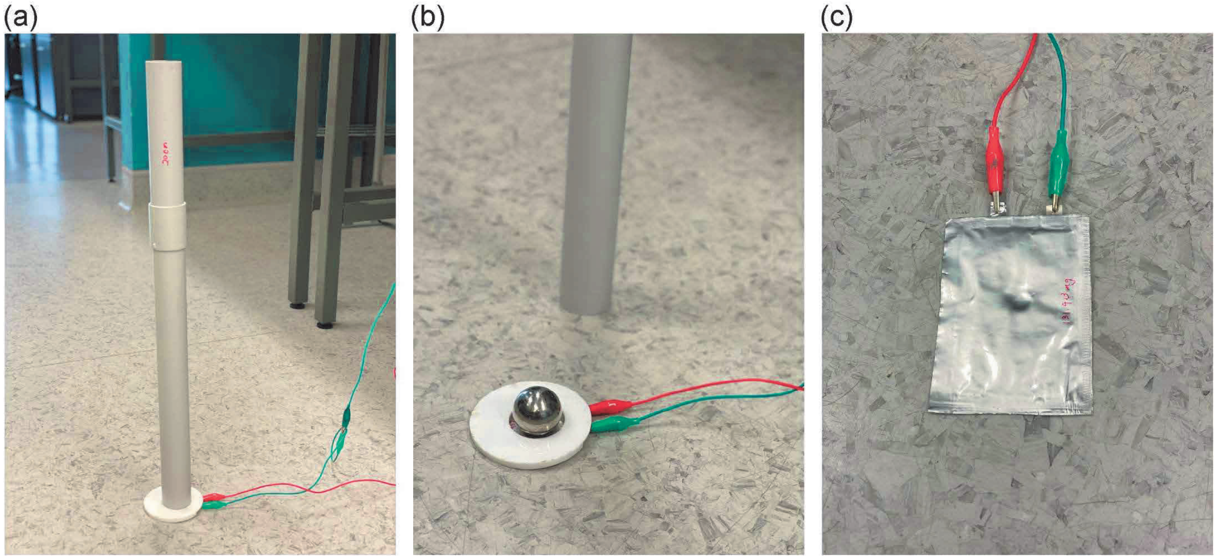

Figure S16. The impact test on pouch cells: (a) set-up; (b) status of the ball dropped onto the cell; (c) the appearance of pouch cell after the impact test.

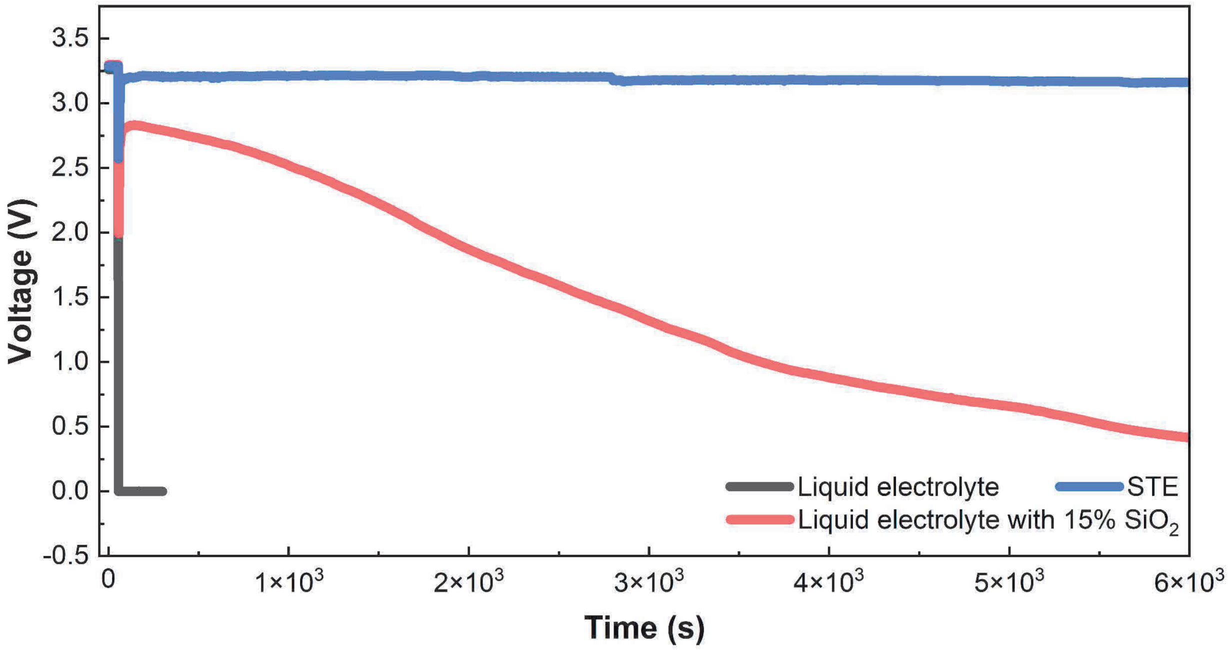

Figure S17. The voltage response of Li-ion pouch cells with different electrolytes of liquid electrolyte, STE, and liquid electrolyte containing 15% SiO_2 under an impact energy of 2.06 J during the period of 6,000s.

Supplementary Tables:

Table S1. Initial viscosity and density of PC solvent and the dispersion containing PEO and silica.

PC solvent containing fillers amount	Initial viscosity (Pa·s)	Density (g/ml)
-	2.3×10^{-3}	1.2
4.2 wt% (0.2 wt% PEO + 4 wt% SiO_2)	86.3	1.24
9.1 wt% SiO_2	158.6	1.26
15 wt% SiO_2	296.5	1.45
20 wt% SiO_2	37,450	1.67

Supplementary Equations:

Equation S1. The equation used for calculating ionic conductivity from the impedance data is as below.[1]

$$\sigma = \frac{d}{R_b \times A} \qquad (1)$$

where bulk resistance (R_b) is interpreted from the impedance data via intercepting the semicircular arc on the Z' axis, d is the distance between electrodes, A is the area of electrolyte.

Equation S2. The equation used for calculating lithium ion transference number,[2] is as follows:

$$t^+ = \frac{I_s(V - I_0R_0)}{I_0(V - I_sR_s)} \qquad (2)$$

where V is the applied voltage, R_0 and R_s are the initial and steady state resistance, and I_0 and I_s are the initial and steady state currents, respectively.

Equation S3. The impact energy can be calculated based on the formula below.[3]

$$E = KE + PE = \frac{1}{2}mv^2 + \frac{1}{2}I\omega^2 + mgh \qquad (3)$$

where the initial velocity (v) is 0 and the angular velocity (ω) is 0 given that the ball is released in static state without any rotation, m is the weight of the ball, g is the gravity of 9.8 m s^{-2}, and h represents the height of the ball's initial position.

References:

[1] Q. Zhu, X. Wang, J. D. Miller, *ACS Appl. Mater. Interfaces* **2019**, *11* (9), 8954.

[2] a) D. R. Franceschetti, J. R. Macdonald, R. P. Buck, *J. Electrochem. Soc.* **1991**, *138* (5), 1368; b) R. Pollard, T. Comte, *J. Electrochem. Soc.* **1989**, *136* (12), 3734; c) K. M. Diederichsen, E. J. McShane, B. D. McCloskey, *ACS Energy Lett.* **2017**, *2* (11), 2563.
[3] C. E. Smith, *J. Appl. Mech.* **1991**, *58* (3), 754.
